# Management of intestinal failure in Europe. A questionnaire based study on the incidence and management

**DOI:** 10.1186/1476-5918-6-7

**Published:** 2007-07-04

**Authors:** Michael Staun, Xavier Hebuterne, Jon Shaffer, Kent V Haderslev, Frederico Bozzetti, Marek Pertkiewicz, Ann Micklewright, Jose Moreno, Paul Thul, Loris Pironi

**Affiliations:** 1 Department of Medical Gastroenterology, Rigshospitalet, Copenhagen, Denmark; 2 Department of Gastroenterology and Clinical Nutrition, Archet University Hospital, Nice, France; 3 Intestinal Failure Unit Hope Hospital, Salford, UK; 4 Department of Surgery, Hospital of Prato, Prato, Italy; 5 Department of Nutrition and Surgery, W. Orlowski University Hospital, Warsaw, Poland; 6 University Hospital, Queen's Medical Centre, Nottingham, UK; 7 Department of Nutricion Clinica Y Dietetica, Hospital 12de Octobre, Madrid, Spain; 8 Department of Surgery, Charité University Hospital, Berlin, Germany; 9 Department of Internal Medicine and Gastroenterology, Center for Chronic Intestinal Failure, St. Orsola-Malpighi Hospital, Bologna, Italy

## Abstract

**Background:**

Intestinal failure is the outcome of a number of gastrointestinal diseases and characterized by significant reduction in functional gut mass. If not resolved patients often face long-term nutritional support. This study gathered information about how patients referred with intestinal failure are managed in specialised European centres.

**Methods:**

A questionnaire was circulated in 7 European countries via representatives of the ESPEN-HAN working group to seek information about experience in treating patients with intestinal failure. We asked about clinical outcome, information about structure and organisation of the department, referral criteria, treatment procedures and guidelines.

**Results:**

17 centres in 6 European countries completed the questionnaire: UK, n = 6, France, n = 4, Spain, n = 3, Denmark, n = 2, Italy, n = 1, Poland, n = 1. The experience of the centres in treating patients was in the range 12–30 years. The total number of patients on HPN in all centres was 590. The number of patients referred to centres with intestinal failure during the period January to December 2000 was n = 882: UK, n = 375 (range 2–175), France, n = 308 (range 24–182), Italy and Spain, n = 43 (range 9–52), Denmark n = 51 (range 14–37), the centre in Poland included 53 patients. Comparing all centres the following distribution among patients (median % (range%)) with regard to the endpoints were reported: Oral nutrition 32% (23–50%), enteral/tube feeding 11% (4–23%), HPN 36% (15–57%), lost to follow up 10% (0–35%), dead 9% (5–18%). No patients had an intestinal transplant.

**Conclusion:**

The study provides information about how patients with intestinal failure are managed across Europe and the data indicates that treatment practice varies between countries.

## Background

Intestinal failure is defined by a reduction in the functional gut mass to the level that the patient depends on parenteral supply of nutrients, water and electrolytes to survive [1]. The condition is not an uncommon endpoint for a variety of diseases including Crohn's disease, complications following surgery, mesenteric vascular disease and radiation enteritis [2-4]. Intestinal decompensation may resolve quickly or persist as a chronic condition and the patient may then face long-term nutritional support [5,6]. Most often, patients or relatives are trained to manage procedures and treatment can then be offered in the home as *home parenteral nutrition *(HPN).

The studies of the ESPEN-HAN Working group and others have provided knowledge about the indication, incidence, prevalence and the prognosis of patients managed on home parenteral nutrition [7,8]. However, data on the incidence and prevalence as well as management of patients with intestinal failure, from which the HPN-group generally is recruited, are rather scarce and little is known about the outcome of these patients. Clinically, patients with intestinal failure cannot be fed by enteral route due to intestinal complications or active disease and most patients need parenteral nutritional supply. The indication for referral to specialised centres is specific clinical problems such as assessment of short bowel, management of fistula or sepsis, venous access problems and the need of specialised abdominal surgery.

The aim of the present study was to obtain information about how centres in Europe manage patients with intestinal failure. Using a questionnaire, we gathered information about experience, structure and organisation of the departments involved, about specific aspects of treatment procedures, existing guidelines, referral criteria, and final outcome for patients with intestinal failure.

## Methods

### The design of the questionnaire

The ESPEN-HAN Working group designed the questionnaire for the study. The questions covered the following: Experience with treatment of these patients, including number of years with experience on home parenteral nutrition. Also, we asked for information about the number of patients referred during the year 2000 and information about endpoints of treatment, enteral nutrition, parenteral nutrition and length of stay in the hospital. Furthermore, we collected information about the structure of the department with relation to treatment of patients with intestinal failure, the number of beds for management of intestinal failure, the presence of a nutrition team and specialized teams for management of central lines, experience with intestinal transplantation and research within in this specific group of patients. The pattern of referral of these patients was also investigated as well as the level of information available to referring departments. Furthermore, departments were asked about treatment procedures, education of personnel in the management of intestinal failure and we asked for information about written guidelines.

### The selection of centres for the study

This study was carried out in selected centres, defined as centres with a special interest and experience in the management of these patients, but no specific criteria for the selection of centres were defined. Thus, the questionnaire was circulated to the centres through the individual ESPEN-HAN group members of the European countries. A covering letter describing the purpose of the study was sent to each centre from the ESPEN-HAN group member.

### Statistics

All data are givens as median and range unless otherwise indicated.

## Results

### Centres and patients

Table 1 shows the number of centres in each country invited to participate in the investigation, the number of responders and the total number of patients referred with intestinal failure during the study period from January 1^st ^to December 31^st ^2000. A total of 23 centres across Europe were invited to participate and 17 responded. These centres reported that a total of 882 patients were referred during the period of investigation. For the previous year of 1999 centres reported a total number of 564 referred with intestinal failure.

**Table 1:** Number of centres invited, number of responding centres and the number of patients referred with intestinal failure in 2000.

**Country**	**Number of centres invited**	**Number of centres that responded**	**Patients referred with intestinal failure from January to December 2000**
UK	8	6	375
France	6	4	308
Italy	1	1	52
Poland	1	1	53
Spain	6	3	43
Denmark	2	2	51
Total	23	17	882

### Structure and experience of centres

Centres answered that the total number of beds reserved solely for treatment of intestinal failure were 111, and within each country the number of beds ranged from 0–42. Centres had a mean experience of 16 years (range 12–30) in the management of patients with intestinal failure and currently had 590 patients on HPN due to chronic intestinal failure. Of the centres, 33% were specialised in medical gastroenterology, 33% in surgical gastroenterology and 33% had both disciplines within the same unit. Of the centres 5 (29%) answered that they were engaged in research projects within this field. One centre had experience with intestinal transplantation, 3 centres (17%) answered that they had some experience with this procedure, and the remaining 76% had no experience with intestinal transplantation. Table 2 shows the total number of physicians and nurses involved in the treatment of these patients in all centres. Table 3 shows the number of hours spent per week by doctors and nurses on the task of taking care of patients with intestinal failure. The table indicates that there are significant differences in how the treatment of these patients is organized. The number of hours spent per week by doctors for each country ranged from 22 to 375 and was not related to the number of beds assigned for this treatment. When comparing time spent per bed the range was broad for both nurses and physicians.

**Table 2:** Number of physicians and nurses involved in treatment of intestinal failure in each country.

	**Physicians**		**Nurses**	
***Country***	***n***	***% of total staff***	***n***	***% of total staff***
UK	9	24	36	49
France	8	22	15	20
Italy	1	3	3	4
Spain	10	27	12	16
Poland	4	11	2	3
Denmark	5	14	6	8
Total	37		74	

**Table 3:** Resources used by nurses and physicians reported by centres as hours per week.

		**Physician**			**Nurse**		
			
***Country***	***Number of beds for ITF treatment***	***Hours/week***	***% Of total***	***Hours/week/bed***	***Hours/week***	***% Of total***	***Hours/week/bed***
UK	42	22	3	0.52	1325	59	31.5
France	29	151	21	5.2	169	8	5.8
Italy	2	24	3	12.0	3	0	1.5
Spain	0	375	51	-	469	21	-
Poland	0	54	7	-	48	2	-
Denmark	38	105	14	2.7	218	10	5.7
Total	111	731			2232		

### Referral of patients and organization of treatment

The centres were asked if they believed that all patients diagnosed with intestinal failure were being referred for treatment when required. Two centres (11%) in Spain and Poland reported that all patients were referred. Of the centres n = 8 (47%) were of the opinion that more than 75% of patients were referred for treatment and 2 centres (11%) in France and Spain answered that they believed that no more than 50% of patients were being referred for treatment. Five centres (31%) in the UK and Denmark were not sure what to answer.

Centres were asked about pattern of referral criteria, geographical and speciality. Centres were allowed more than one answer. Of the centres, 11 had patients referred from the region, 7 centres received patients referred more randomly. The departments of abdominal surgery referred the majority of patients for treatment. All centres except one received patients from this speciality and 7 centres received patients from departments of medical gastroenterology. Of the 17 centres, 4 (24%) were reimbursed for their services and 6 (34%) centres had a waiting list.

Centres were asked if they believed that the treatment of intestinal failure was well organized in their country or region. Of the centres, 35% reported that treatment was well organized and 65% answered that organization needed improvement.

### Referral criteria

Departments were asked if a specific set of referral criteria were used and if so, we asked who had defined these criteria. Of the centres 7 (41%) reported to adhere to guidelines of referral defined by the department or the hospital.

### Information about the service provided to referring departments

With regard to the treatment of intestinal failure, centres were asked if the clinical director/head of department initiated activities to inform about the experience and 'know how' of the department. Specific questions asked for workshops about management of intestinal failure at the institution, if the 'know how' were accessible on a web site and whether any written information about the unit was available on demand. Of the centres 8 (47%) answered that the head of department took initiatives to inform about the service provided and 5 centres (29%) reported that workshops were being held on intestinal failure management. In 7 (41%) centres written information was available on request.

### Guidelines

We asked for information about written guidelines on treatment of intestinal failure in general and guidelines for parenteral and enteral nutrition. Of the responders, 5 centres (29%) had written guidelines on how to manage intestinal failure. All centres except one had guidelines for parenteral nutrition and 13 (76%) had guidelines for enteral nutrition. With regard to management of intestinal failure, we asked more specifically for information about guidelines on some of the most frequent complications and clinical difficulties associated with this clinical condition: sepsis and catheter related sepsis, fistula management, high output stoma, vein thrombosis and liver disease. The results are shown in Table 4.

**Table 4:** Number of centres with written guidelines for management of guidelines for complications/clinical issues associated with intestinal failure.

***Complication***	***n***	***% of all***
Sepsis	12	71
Fistula	3	18
Catheter related sepsis	17	100
Catheter related venous thrombosis	10	59
High output stoma	9	53
Liver disease	3	18

### Length of hospital stays for patients with intestinal failure

Table 5 shows the median and range of the length of the hospital stay. Data are pooled for each of the countries involved in the study.

**Table 5:** Length of hospital stays.

	***Median (days)***	***Range (days)***
UK	79	2–700
France	71	1–210
Italy	17	5–93
Spain	85	10–150
Poland	25	9–86
Denmark	228	7–360

### The endpoints of treatment of intestinal failure

Centres were asked for data on endpoints of treatment for patients admitted during the period January 1^st ^to December 31^st ^2000, and discharged before December 31^st ^2000. The following endpoints were considered: Discharged from hospital with oral or enteral nutrition or tube feeding and HPN. Also we asked for data on death rate, intestinal transplantation performed and whether patients were lost to follow up. Table 6 shows the main results for all centres with regard to endpoint of treatment. The table was compiled by the inclusion of data from all centres.

**Table 6:** The endpoints of treatment of intestinal failure for all patients in the study.

	***Median (%)***	***Range (%)***
Oral nutrition	32	23–50
Enteral nutrition/tube feeding	11	4–23
HPN	36	15–57
Died	9	5–18
Intestinal transplantation performed	0	0
Lost to follow up	10	0–35

## Discussion

Seventeen out of 23 centres responded and a possible bias is that centres with a known expertise may not have been asked to participate or may not have responded and this should be considered when evaluating the data. Data are representative in the sense that information was obtained from across Europe involving 17 centres in 6 countries and the survey includes about 900 patients diagnosed with intestinal failure. The number of centres in each country varied significantly and was not correlated to number of inhabitants, but more likely reflects the organization of treatment of these patients on a national level.

Epidemiological data about the prevalence or incidence of intestinal failure in adults or children are not available. This possibly reflects that the clinical entity has a low prevalence with a multifactorial aetiology. In addition, criteria for this diagnosis have never firmly been agreed upon. The prevalence of home parenteral nutrition has been reported, but these data do not include patients with intestinal failure with a temporary need for parenteral support. Surveys on HPN in Europe indicated an incidence of 2–3 patients per million and the prevalence was reported to about 4 per million with a broad range [7,8]. No studies on prevalence and incidence of HPN in Europe have been published recently since the organisation of this service in many countries has changed from being centralised. In the US larger figures have been reported possibly reflecting that the indication for this treatment differs between continents [9,10]. The organizational structure of HPN in Europe has changed in the last few years but the most recent survey does not provide data that can be used for calculation of prevalence and incidence of HPN on a national level, at least only in some countries (11). This makes it even more difficult to estimate epidemiological data for intestinal failure since a varying number of patients are unaccounted for, as in this study, or may be weaned off parenteral nutrition before entering HPN programs. In keeping with this, Messing *et al*. [5] reported that as many as 27% of patients with short bowel syndrome were off parenteral nutrition one month after the event that caused intestinal failure.

The survey confirms that patients with intestinal failure stay in hospital for significant amount of time, hence, up to 700 days in a UK centre, and the median length of stay ranges between 17 in Italy and 228 in Denmark. This is not surprising; intestinal failure is often associated with a number of additional clinical problems such as high output stoma, fistula, sepsis and liver and kidney failure and patients may need specialized treatment for a long period. We are not aware of previous data on length of stay for this group of patients.

The large variation in mortality rate reported between countries could be related to a greater number of cancer patients being treated in some centres. Surveys in Europe have shown that cancer is a more common indication for HPN in the southern part of Europe [11].

The clinical challenge is to re-establish intestinal function whenever possible so that patients with intestinal failure can be managed on oral or enteral nutrition. In this study we found that about 43% of the patients could be send home on oral or enteral nutrition. About on third of the patients were discharged from hospital with parenteral nutrition. Interestingly, none of the centres had referred patients for intestinal transplantation procedures in accordance with a recent study on the indication for intestinal transplantation showing that European centres have some reluctance to refer patients for this procedure [12].

Experience of the centres participating in this study varies significantly, but all centres had an experience of 12 years or beyond and were handling a considerable amount of patients with chronic intestinal failure maintained on HPN. Management of patients with intestinal failure in many cases requires a close collaboration between surgical and medical gastroenterology and the centres reported that patients were treated either in the individual speciality or in a unit with access to both disciplines. Resources in terms of time spent on these patients by nurses and physicians showed considerable variation with the highest values reported for both nurses and physicians reported in the UK and Denmark. In the UK, intestinal failure units have been established and in Denmark treatment of intestinal failure patients has been assigned to very few centres.

We asked for information about referral of patients with intestinal failure and the answers were diverging. Only 10% of centres were of the opinion that all patients were being referred, but close to 60% of the centres in different countries were quite certain that no more than 50–75% of the intestinal failure patients were being referred for treatment. The results indicate that treatment is not well organized in all countries and that may not even be the case in countries with a set of national guidelines for treatment. This is also reflected in the referral pattern, patients are reported coming from both the region in which the centre is placed and patients are also sent from other departments. Although centres did not believe that all patients were referred for treatment about one third had a waiting list. The majority of centres were of the opinion that treatment could be better organized.

Guidelines for treatment of intestinal failure have not been developed in all centres and only 29% reported that such guidelines were available. With regard to specific treatment with enteral and parenteral nutrition, the majority of centres had guidelines. The management of the most common complications related to intestinal failure was also described in guidelines supporting that centres generally were highly specialised in taking care of these patients.

## Conclusion

This study indicates that patients with intestinal failure are being managed across Europe in specialised units in the setting of both surgical and medical gastroenterology; the organizational structure of this service varies significantly between centres. The study reports on about 900 patients diagnosed with this diagnosis and shows that about 43% are discharged with per oral or enteral nutrition and 36% with parenteral nutrition. Mortality rate during a one-year observation period is about 10% and no patients were referred for intestinal transplantation. Not all patients are referred for treatment and not all centres have specific guidelines for management of intestinal failure, although they in general have much experience. More information about epidemiology of intestinal failure can provide a better understanding of disease impact. This study does not provide details about outcome and prognosis in relation to primary disease and complications; this would require a prospective study in selected centres.

## Authors' contributions

MS conceived of the study, and participated in its design and coordination, and performed the first draft of the manuscript. All authors contributed collection of data and approved the final manuscript.

## Declaration of competing interests

The author(s) declare that they have no competing interests.

## Appendix

We are indebted to contributors to HPN survey in Europe, (see Table 7).

**Table 7:** Appendix

**Country**	**Principal investigator**
United Kingdom	Professor CR Pennington (Dundee)
United Kingdom	SJ Middleton (Cambridge)
United Kingdom	K. Fearon (Edinburgh)
United Kingdom	Helen Hamilton (Oxford)
United Kingdom	K.A Forbes (Harrow)
France	Dr. Cecile Chambrier (Lyon)
France	Bernard Messing (Paris)
France	Jean-Marie Reimund (Strasbourg)
Spain	Jose M Moreno/MIguel Leon Sanz (Madrid)
Spain	Dr. Pilar Garcia Peris (Madrid)
Denmark	Henrik HØjgaard Rasmussen (Aalborg)
